# Perceived reward adequacy as a key mechanism for retaining medical doctors in health systems: A structural equation modelling study

**DOI:** 10.4102/phcfm.v18i1.5381

**Published:** 2026-08-06

**Authors:** Seth Fenyi, Solomon Keelson

**Affiliations:** 1 Department of Business Administration, Faculty of Administration, Accra Institute of Technology, Accra, Ghana; 2 Department of Administration, Faculty of Management, Takoradi Technical University, Takoradi, Ghana

**Keywords:** total rewards, reward adequacy, reward expectation, medical doctor retention, Ghana, structural equation modelling

## Abstract

**Background:**

Retaining medical doctors remains a major challenge for health systems, particularly in low- and middle-income countries (LMICs), where workforce instability undermines continuity of care and service delivery. Although total rewards are widely used to improve retention, it remains unclear whether doctors stay because rewards are available or because they perceive them as fair and adequate.

**Aim:**

To examine whether reward adequacy and reward expectation mediate the relationship between total rewards and medical doctor retention.

**Setting:**

The study was conducted in public healthcare facilities in Ghana.

**Methods:**

A quantitative cross-sectional survey was conducted among 400 medical doctors recruited through stratified facility-level sampling. Data were collected using a structured questionnaire and analysed using Statistical Package for the Social Sciences (SPSS) version 26 and SmartPLS version 4. Partial least squares structural equation modelling assessed measurement validity and bootstrapped direct and indirect effects.

**Results:**

Total rewards had no significant direct effect on retention intention (*β* = 0.048, *p* = 0.507). Total rewards positively influenced reward adequacy (*β* = 0.689, *p* < 0.001), and reward adequacy positively influenced retention intention (*β* = 0.623, *p* < 0.001). Reward adequacy significantly mediated the total rewards–retention relationship (*β* = 0.429, *p* < 0.001), whereas reward expectation did not.

**Conclusion:**

The retention value of total rewards depends primarily on doctors’ perceptions of adequacy, fairness and sufficiency. Health systems should prioritise transparent and equitable reward structures rather than relying solely on financial and non-financial incentives.

**Contribution:**

The study identifies reward adequacy as the principal mechanism through which total rewards influence medical doctor retention in an LMIC primary healthcare context.

## Introduction

Retention of medical doctors is a persistent concern for health systems, particularly in low- and middle-income countries (LMICs), where physician shortages undermine continuity, accessibility and quality of care.^1,2^ In this article, the terms medical doctors and physicians are used interchangeably to refer to fully qualified doctors providing clinical services, excluding interns and temporary clinical trainees.

Turnover among doctors can increase waiting times, disrupt patient–provider relationships, reduce institutional memory and place additional workload pressure on remaining staff, especially in primary health care facilities and district hospitals.^3,4^ A stable medical workforce is therefore central to resilient primary health care delivery and to the broader performance of health systems.

Total rewards are often used to strengthen workforce retention. They represent a holistic reward approach that combines financial rewards, such as salaries and allowances, with non-financial rewards, including promotion opportunities, career development, recognition and work–life balance.^5,6^ In health systems, total rewards are intended to attract doctors, strengthen commitment and reduce movement to other sectors or countries.^7,8^

However, the literature does not consistently show that the presence or improvement of rewards automatically translates into retention. Some studies report positive associations between financial and non-financial rewards and doctors’ intention to stay, while others show that remuneration alone may have limited retention value when workloads remain high, promotion systems are unclear or organisational support is weak.^4,9,10^ This inconsistency suggests that retention may depend not only on the objective availability of rewards but also on how doctors interpret and evaluate those rewards.

Two perceptual mechanisms are relevant to this issue. Reward adequacy refers to the extent to which doctors perceive their total rewards as fair, sufficient and commensurate with workload, responsibilities and professional contribution.^11,12^ Reward expectation refers to doctors’ anticipation of future rewards, such as salary progression, promotion, training and career development opportunities, based on past experiences and organisational practices.^13,14^

The rationale for this study is that health systems may fail to retain doctors when reward policies are treated only as administrative inputs rather than as perceived indicators of fairness, recognition and organisational commitment. Evidence on how reward adequacy and reward expectation mediate the total rewards-retention relationship remains limited in Ghana and similar LMIC primary health care contexts. Addressing this gap is important for designing reward systems that are not only available on paper but also experienced by doctors as fair, credible and sufficient.

This study therefore examined whether reward adequacy and reward expectation mediate the relationship between total rewards and medical doctor retention. The objective was to determine whether total rewards influence retention directly or indirectly through doctors’ perceptions of present adequacy and future reward expectations.

## Theoretical foundation and hypotheses

The study was guided by equity theory and expectancy theory. Equity theory proposes that employees evaluate rewards by comparing what they receive with their inputs and with the rewards received by relevant referents.

Applied to doctors, reward systems may influence retention when doctors perceive that remuneration, promotion and development opportunities are fair relative to their workload, responsibilities and professional contribution. Expectancy theory suggests that motivation and behavioural intention are shaped by beliefs that effort and continued organisational membership will lead to valued outcomes. Thus, retention may also depend on whether doctors believe future rewards are credible and attainable.

Based on these perspectives, total rewards were expected to influence retention both directly and indirectly. Firstly, better total rewards may increase doctors’ intention to remain in service. Secondly, total rewards may improve perceived reward adequacy, which should in turn strengthen retention. Thirdly, total rewards may influence reward expectation, although this pathway may be weaker in settings where future promotions and reward adjustments are uncertain:

**H1:** Total rewards are positively associated with medical doctor retention.

**H2:** Total rewards are positively associated with reward adequacy.

**H3:** Total rewards are associated with reward expectation.

**H4:** Reward adequacy is positively associated with medical doctor retention.

**H5:** Reward expectation is positively associated with medical doctor retention.

**H6:** Reward adequacy mediates the relationship between total rewards and medical doctor retention.

**H7:** Reward expectation mediates the relationship between total rewards and medical doctor retention.

## Research methods and design

### Study design

A quantitative, cross-sectional survey design was used. The design was appropriate for assessing doctors’ perceptions of rewards, perceived adequacy, reward expectations and retention intention at one point in time. Because the design was cross-sectional, the structural model tested hypothesised theoretical relationships and indirect effects; it did not establish temporal or definitive causal relationships.

### Study setting and participants

The study was conducted among medical doctors working in public healthcare facilities in Ghana. Facilities represented three broad service levels: Primary-level facilities, including clinics and health centres where medical doctors provide primary care or supervisory clinical services; district and secondary hospitals and tertiary hospitals. These levels were selected because they reflect key points in the public health system where doctor retention affects continuity of care, referral quality and service delivery.

Eligible participants were fully qualified medical doctors who were actively involved in clinical service delivery and had worked long enough in the public health system to have experience of organisational reward practices. Doctors on internship, in temporary placement, on leave without regular clinical service or not directly involved in clinical care were excluded.

### Sample size, sampling and recruitment

A stratified sampling approach was used to improve representation across healthcare facility levels. The strata were primary-level facilities, district and secondary hospitals and tertiary hospitals. Lists of eligible doctors were obtained through professional and facility-level networks, and recruitment links were circulated proportionately across the strata. Within each stratum, eligible doctors were invited electronically and were allowed to participate only once. Participation was voluntary, and no identifying information was collected.

The minimum sample size was assessed using statistical power and PLS-SEM requirements. For a medium effect size (*f*^2^ = 0.15), alpha of 0.05, statistical power of 0.80 and a maximum of three predictors pointing to an endogenous construct, the minimum sample required was below the final achieved sample. In addition, PLS-SEM guidance recommends considering model complexity, indicator reliability and bootstrapping precision rather than relying only on the traditional ten-times rule.^15,16^ The final sample of 400 valid responses was therefore considered adequate for estimating the measurement and structural models.

A total of 410 doctors were invited, of whom 400 submitted complete and usable questionnaires, giving a response rate of 97.6%. Records with substantial missing responses, duplicate entries or straight-line response patterns were excluded before analysis.

### Data collection instrument

Data were collected using a structured, self-administered electronic questionnaire between August 2025 and November 2025. The instrument had two sections. Section A captured demographic and work-related characteristics, including age, sex, years of professional experience and facility level. Section B measured the latent constructs using adapted items from previously validated reward, reward perception and retention intention scales.^9,11,13,17^ The items were adapted to fit the Ghanaian public healthcare context.

Total rewards were measured as a higher-order construct reflecting financial rewards and non-financial rewards. Financial rewards captured salary and allowances, while non-financial rewards captured promotion and career development opportunities. Reward adequacy measured perceived fairness, sufficiency and fit between rewards and doctors’ workload, responsibility and contribution. Reward expectation measured doctors’ expectations of future salary progression, allowances, promotion and career development. Medical doctor retention was measured through intention to remain in the current health system or organisation. All scale items were measured on a five-point Likert scale ranging from 1 (strongly disagree) to 5 (strongly agree).

A pilot test was conducted with 20 doctors who were not included in the final study sample. The pilot assessed clarity, completion time, contextual relevance and item wording. Minor wording revisions were made to improve clarity without changing the conceptual meaning of the validated items.

### Data management and preliminary screening

Data were screened before model estimation. Cases with extensive missing data were removed. For retained cases, item-level missingness was checked and was minimal; therefore, complete-case analysis was applied for the final 400 responses. Response patterns were inspected for duplicate submissions and straight-line responses. Descriptive statistics, skewness and kurtosis were examined to assess distributional characteristics, while boxplots and standardised scores were used to inspect potential outliers. Because PLS-SEM is variance based and does not require multivariate normality, it was considered appropriate for the data and the model’s objective.^15^

### Data analysis and structural equation modelling procedure

Data were analysed using Statistical Package for the Social Sciences (SPSS) version 26 (IBM Corp., Armonk, New York, United States [US]) for descriptive statistics and SmartPLS version 4 (SmartPLS GmbH, Monheim am Rhein, Germany) for partial least squares structural equation modelling (PLS-SEM). The analysis followed a two-stage procedure. Firstly, the measurement model was assessed to determine whether the latent constructs were measured reliably and validly. Indicator loadings of 0.70 or higher were preferred although loadings between 0.60 and 0.70 were retained where construct reliability and content validity were adequate. Internal consistency was assessed using Cronbach’s alpha and composite reliability, with values of 0.70 or higher considered acceptable. Convergent validity was evaluated using average variance extracted (AVE), with AVE of 0.50 or higher indicating that the construct explained at least half of the variance in its indicators.

Discriminant validity was assessed using the Fornell–Larcker criterion and the heterotrait-monotrait ratio (HTMT). For the Fornell–Larcker criterion, the square root of each construct’s AVE was expected to exceed its correlations with other constructs. Heterotrait-monotrait ratio values below 0.85 were interpreted as evidence of discriminant validity. Multicollinearity was evaluated using the variance inflation factor (VIF), with VIF values below 5.00 considered acceptable.

Secondly, the structural model was assessed by examining path coefficients, standard deviations, *t*-statistics, *p*-values, coefficient of determination (*R*^2^) and mediation effects. Bootstrapping with 5000 resamples was used to assess the statistical significance of direct and indirect effects. The hypothesised paths were: Total rewards to retention, total rewards to reward adequacy, total rewards to reward expectation, reward adequacy to retention and reward expectation to retention. Mediation was assessed by comparing the total effect, direct effect and bootstrapped specific indirect effects through reward adequacy and reward expectation.

Model fit was considered using the standardised root mean square residual (SRMR), while explanatory power was interpreted using *R*^2^ values for endogenous constructs. Given the predictive and theory-testing objective of the study, the main interpretation focused on reliability, validity, path coefficients, *R*^2^ and bootstrapped indirect effects, consistent with PLS-SEM guidance.

### Ethical considerations

Ethical clearance to conduct this study was obtained from the Topview Medical Centre Research and Ethics Oversight Committee (TMC–REOC) with reference number TMC/ERC/2026/01. Participation was voluntary. Written informed consent was obtained electronically before the questionnaire could be completed. Participants were informed about the study’s purpose, confidentiality, anonymity, voluntary participation and the right to withdraw before submission. No names, staff identification numbers or facility-specific identifiers were collected in the dataset used for analysis.

## Results

### Participant characteristics

A total of 400 valid responses were included in the analysis. The demographic profile showed variation in sex, age, years of experience and facility level, supporting interpretation across different categories of doctors in the public health system. Table 1 summarises the participant characteristics.

**TABLE 1:** Participant characteristics.

Characteristic	Category	*n*	%
Sex	Male	234	58.5
	Female	166	41.5
Age (years)	< 25	11	2.8
	26–35	193	48.2
	36–45	153	38.2
	46–55	43	10.8
	≥ 56	0	0.0
Years of experience	< 5	152	37.9
	5–10	140	35.0
	11–15	37	9.2
	16–20	52	13.1
	> 20	19	4.8
Facility level	Primary-level facility	209	52.3
	District/secondary hospital	105	26.2
	Tertiary hospital	86	21.5

Table 1 shows that the sample comprised 400 medical doctors. Males constituted the majority of respondents (*n* = 234, 58.5%), while females represented 41.5% (*n* = 166). Most participants were aged 26–35 years (*n* = 193, 48.2%), followed by those aged 36–45 years (*n* = 153, 38.2%), indicating that the sample was largely composed of early- to mid-career doctors.

Regarding professional experience, most respondents had less than 5 years of experience (*n* = 152, 37.9%) or 5–10 years of experience (*n* = 140, 35.0%). This suggests that a substantial proportion of the sample was still within the early stages of their medical careers, making reward adequacy and retention concerns particularly relevant. At the facility level, over half of the respondents worked in primary-level facilities (*n* = 209, 52.3%), followed by district and secondary hospitals (*n* = 105, 26.2%) and tertiary hospitals (*n* = 86, 21.5%).

Overall, the demographic profile reflects a predominantly early- to mid-career medical workforce drawn from different levels of Ghana’s healthcare system, providing an appropriate basis for examining total reward, reward adequacy, reward expectation and physician retention.

### Measurement model assessment

The measurement model showed acceptable reliability and validity, consistent with established PLS-SEM criteria for reflective measurement assessment.^15,16^ Cronbach’s alpha and composite reliability values were above the 0.70 threshold, supporting internal consistency reliability. Average variance extracted values were above 0.50, supporting convergent validity. Variance inflation factor values were below 5.00, indicating that multicollinearity was not a concern. Table 2 presents the reliability, validity and collinearity results.

**TABLE 2:** Construct reliability, convergent validity and collinearity assessment.

Construct	Number of retained items	Cronbach’s alpha	Composite reliability	AVE	VIF range	Decision
Total rewards	4	0.846	0.896	0.684	1.987–2.587	Acceptable
Reward adequacy	5	0.844	0.889	0.619	1.411–2.545	Acceptable
Reward expectation	7	0.938	0.947	0.720	2.186–3.906	Acceptable
Retention intention	5	0.890	0.919	0.696	1.680–3.432	Acceptable

Note: Thresholds applied: Cronbach’s alpha ≥ 0.70; composite reliability ≥ 0.70; AVE ≥ 0.50; VIF < 5.00.

AVE, average variance extracted; VIF, variance inflation factor.

Discriminant validity was supported by the Fornell–Larcker criterion and HTMT. The square root of AVE for each construct exceeded its correlations with other constructs, and all HTMT values were below the recommended 0.85 threshold. These results indicated that the constructs were empirically distinct and suitable for structural model assessment.

In particular, TotalRD demonstrated satisfactory reliability and convergent validity, confirming the adequacy of the total rewards measurement. The retention intention (RET) dimension also showed strong construct validity, with high internal consistency and acceptable AVE, supporting its suitability for assessing retention intention in the structural model.

### Structural model and explanatory power

The structural model explained a meaningful proportion of variance in the endogenous constructs, as shown in Table 3. Total rewards explained substantial variance in reward adequacy, indicating that doctors’ perceptions of adequacy were closely related to the reward system. The variance explained in reward expectation was smaller, suggesting that future-oriented expectations may be influenced by other institutional factors beyond current rewards. The model also explained a meaningful proportion of variance in retention intention, showing that reward-related perceptions contributed to doctors’ stated intention to remain.

**TABLE 3:** Explanatory power of endogenous constructs.

Endogenous construct	*R* ^2^	*R*^2^ adjusted	Interpretation
Reward adequacy	0.475	0.474	Substantial/moderate
Reward expectation	0.064	0.062	Low
Retention intention	0.411	0.406	Moderate

### Direct effects and hypothesis testing

The direct path from total rewards to retention intention was positive but not statistically significant (*β* = 0.048, *t* = 0.663, *p* = 0.507), so H1 was not supported. Total rewards were positively and significantly associated with reward adequacy (*β* = 0.689, *t* = 17.674, *p* < 0.001), supporting H2 and aligning with evidence that employees’ evaluation of reward fairness and sufficiency is central to workforce outcomes.^5,11^ Total rewards were significantly associated with reward expectation (*β* = −0.254, *t* = 4.068, *p* < 0.001), supporting H3 in statistical terms but indicating a negative direction.

**FIGURE 1:**
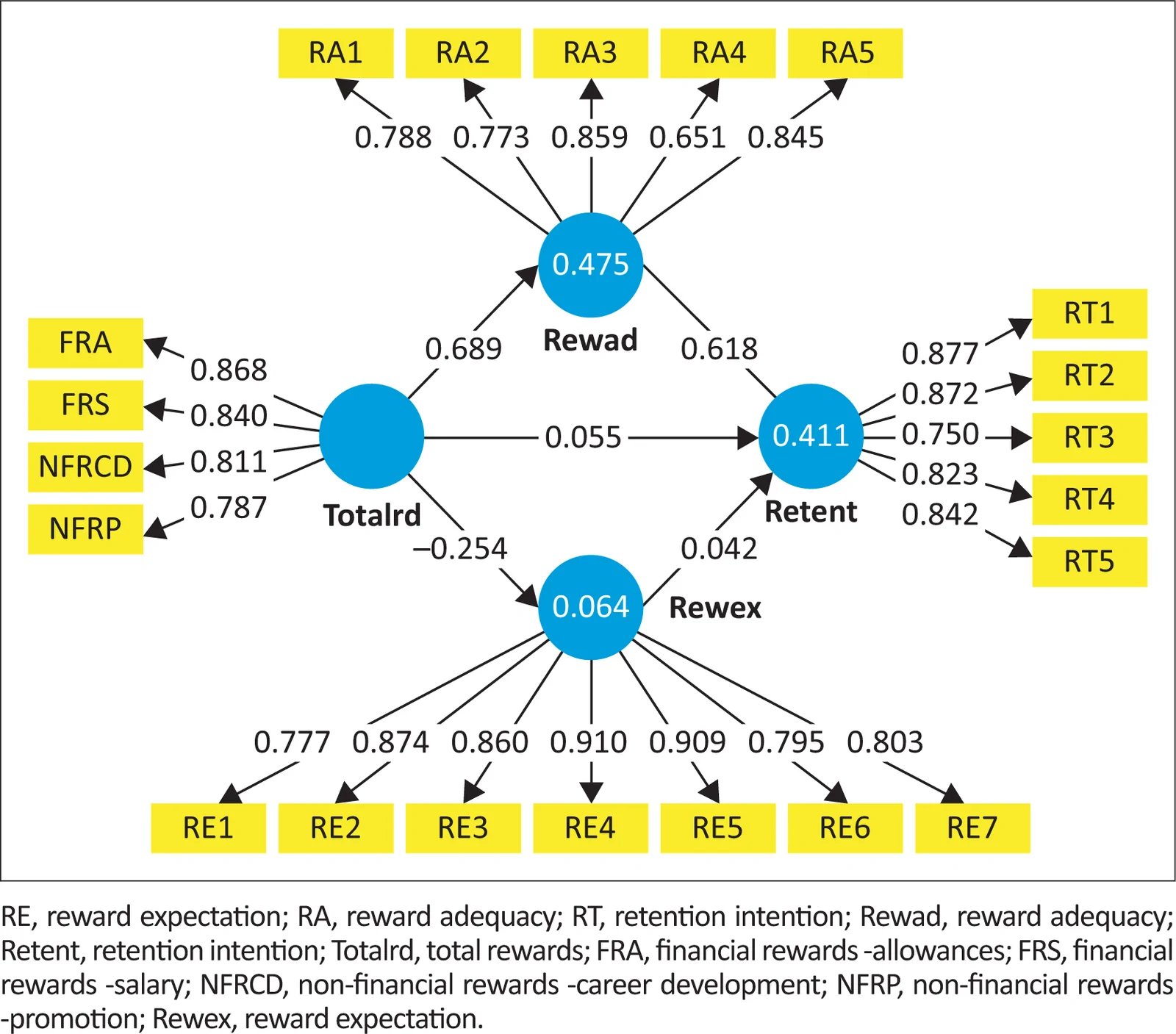
Measurement assessment model result.

Reward adequacy was positively and significantly associated with retention intention (*β* = 0.623, *t* = 11.773, *p* < 0.001), supporting H4 and reinforcing the argument that adequate and fair rewards can strengthen employees’ intention to remain.^6,9^ Reward expectation was positive but not statistically significant (*β* = 0.045, *t* = 1.078, *p* = 0.281), so H5 was not supported. These findings indicate that doctors’ current perception of reward sufficiency was more important for retention intention than expectations of future rewards, Table 4 and Figure 1.

**TABLE 4:** Direct path coefficients and hypothesis testing.

Hypothesis	Structural path	*β*	s.d.	*t*-value	*p*-value	Decision
H1	Total rewards -> Retention intention	0.048	0.072	0.663	0.507	Not supported
H2	Total rewards -> Reward adequacy	0.689	0.039	17.674	< 0.001	Supported
H3	Total rewards -> Reward expectation	−0.254	0.062	4.068	< 0.001	Supported, negative direction
H4	Reward adequacy -> Retention intention	0.623	0.053	11.773	< 0.001	Supported
H5	Reward expectation -> Retention intention	0.045	0.042	1.078	0.281	Not supported

s.d., standard deviation.

### Mediation analysis

The mediation results showed that reward adequacy significantly mediated the relationship between total rewards and retention intention. The total effect of total rewards on retention was significant (*β* = 0.465, *p* < 0.001), but the direct effect became non-significant after reward adequacy and reward expectation were included (*β* = 0.048, *p* = 0.507) (Table 5). The specific indirect effect through reward adequacy was significant (*β* = 0.429, *t* = 8.884, *p* < 0.001), supporting H6 and indicating full mediation.

**TABLE 5:** Specific indirect effects and mediation assessment.

Hypothesis	Mediating path	Total effect		Direct effect		Indirect effect *β*	s.d.	*t*-value	*p*-value	Decision
		*β*	*p*-value	*β*	*p*-value					
H6	Total rewards -> Reward adequacy -> Retention intention	0.465	< 0.001	0.048	0.507	0.429	0.048	8.884	< 0.001	Supported; full mediation
H7	Total rewards -> Reward expectation -> Retention intention	0.465	< 0.001	0.048	0.507	−0.011	0.013	0.893	0.372	Not supported

s.d., standard deviation.

The indirect effect through reward expectation was not significant (*β* = −0.011, *t* = 0.893, *p* = 0.372), so H7 was not supported. This result indicates that future-oriented reward expectations did not explain the relationship between total rewards and retention intention in this model.

## Discussion

This study examined whether reward adequacy and reward expectation explain the relationship between total rewards and medical doctor retention in a Ghanaian public health system context. The key finding is that total rewards did not directly predict retention intention after the mediators were included. Instead, total rewards influenced retention primarily through reward adequacy. This supports the argument that reward policies retain doctors when they are experienced as fair, sufficient and aligned with professional contribution, rather than merely because rewards are formally available.

The non-significant direct relationship between total rewards and retention suggests that salary, allowances, promotion and career development opportunities may not be sufficient on their own to stabilise the medical workforce. This is consistent with evidence that retention is shaped by how healthcare workers evaluate rewards in relation to workload, career prospects and organisational support.^4,9,10^ In primary health care and district hospital contexts, doctors may continue to feel undervalued when rewards are not perceived as commensurate with workload intensity or professional responsibility.

Reward adequacy was the strongest mechanism in the model. This finding is theoretically consistent with equity theory because doctors are likely to compare their contributions and rewards with those of peers within the health system and with alternative opportunities outside the system. It also aligns with prior evidence that perceived adequacy, fairness and sufficiency of rewards are important predictors of employee outcomes and retention-related attitudes.^11,12^ When rewards are perceived as fair and sufficient, doctors may be more willing to remain despite resource constraints. Conversely, perceived inadequacy may strengthen dissatisfaction, disengagement and intention to leave, particularly in health systems where workload pressure and organisational support influence retention decisions.^4,10^

Reward expectation did not significantly mediate the relationship between total rewards and retention. One explanation is that future rewards may be perceived as uncertain in settings where promotion timelines, salary progression and career development opportunities are not consistently communicated. This interpretation is consistent with expectancy theory, which suggests that motivation and retention-related intentions depend partly on whether employees believe future organisational rewards are credible, attainable and linked to continued contribution.^13,14^ Doctors may therefore base retention decisions more on current reward adequacy than on future reward promises. This finding suggests that policy credibility and transparent implementation may be necessary before future-oriented rewards can influence retention.

The significant negative association between total rewards and reward expectation is an important quantitative finding. It suggests that the existence or improvement of current reward packages may not automatically raise doctors’ expectations of future rewards when salary progression, allowances, promotion and career development opportunities are perceived as uncertain or inconsistently implemented. This finding extends expectancy-based interpretations of retention by showing that future-oriented reward expectations may depend not only on the availability of rewards but also on the credibility, transparency and consistency of reward-policy implementation.^13,18^ It also supports the broader argument that total rewards contribute more meaningfully to retention when they are experienced as fair, adequate and reliably implemented, rather than merely present as formal organisational policies.^6,9,17^

### Implications for primary health care and health policy

For primary health care delivery, the findings imply that retention strategies should prioritise fairness, sufficiency and transparency in reward systems. High doctor turnover can weaken continuity of care, reduce trust between patients and providers and increase pressure on remaining staff. Improving perceived reward adequacy may therefore contribute indirectly to service continuity and system resilience.

At the policy level, national and facility-level managers should move beyond periodic salary adjustments and evaluate how doctors perceive the full reward package. Practical strategies include transparent criteria for allowances, clear promotion pathways, consistent access to career development, timely communication of reward policies and routine assessment of perceived reward adequacy. These strategies may be particularly relevant in LMIC contexts where fiscal constraints limit continuous increases in monetary rewards.

### Strengths and limitations

A strength of the study is its focus on the perceptual mechanism through which total rewards influence retention, using PLS-SEM to assess both measurement quality and structural relationships. The sample size of 400 also provided adequate statistical power for estimating the model. However, the cross-sectional design limits causal interpretation. The results should therefore be interpreted as evidence of hypothesised theoretical associations rather than proof of causality. The use of self-reported data may also introduce common method bias although anonymity and electronic self-administration may have reduced social desirability pressure. Finally, the model focused on reward-related factors and did not include other potential determinants of retention, such as leadership, workload, organisational support or migration intention.

### Recommendations for future research

Future studies should use longitudinal designs to assess whether changes in reward adequacy predict actual retention over time. Qualitative research could also explore how doctors define adequate rewards in different facility levels and career stages. Further studies may test broader models that include workload, burnout, organisational support, leadership and migration intention alongside reward adequacy.

## Conclusion

The study concludes that total rewards influence medical doctor retention primarily through perceived reward adequacy. In the Ghanaian public health system context, doctors’ intention to remain appears to depend less on the mere existence of financial and non-financial rewards and more on whether those rewards are experienced as fair, sufficient and aligned with professional contribution. Reward expectation did not significantly mediate the relationship, suggesting that uncertain future rewards may have limited retention value unless current rewards are perceived as adequate. Health systems seeking to retain doctors should therefore design transparent, equitable and consistently implemented reward structures that strengthen perceived adequacy and support workforce stability.
